# Exploring the “Two-Hit” Phenomenon of Guillain-Barré Syndrome Following Respiratory Syncytial Virus and Influenza Vaccinations

**DOI:** 10.7759/cureus.68899

**Published:** 2024-09-07

**Authors:** Kathleen Huynh, Samantha Dion, Mary T Mahoney, Ayfer Ekiz

**Affiliations:** 1 Internal Medicine, Touro College of Osteopathic Medicine, Middletown, USA; 2 Internal Medicine, Garnet Health Medical Center, Middletown, USA

**Keywords:** case report, intravenous immunoglobulin, neuropathy, vaccine coadministration, influenza vaccination, respiratory syncytial virus (rsv) vaccination, guillain-barré syndrome (gbs)

## Abstract

Guillain-Barré syndrome (GBS) is a neurological disorder characterized by peripheral, autoimmune-mediated demyelinating polyneuropathy, which can cause muscle weakness and paralysis. While most cases are triggered by respiratory or gastrointestinal infections, vaccinations have also been linked to GBS pathogenesis. The association of the influenza vaccine and GBS, notably prevalent during the 1976 United States swine flu pandemic, has significantly decreased with contemporary seasonal influenza vaccines. At the same time, cases of GBS have been reported with newer vaccines, like the recently approved respiratory syncytial virus (RSV) vaccines. However, their exact relationship with autoimmune demyelinating polyneuropathy remains unknown. In this report, we present a case of a 60-year-old man who developed GBS two weeks after receiving the new Pfizer's RSV vaccine in conjunction with the influenza vaccine for the first time.

## Introduction

Guillain-Barré syndrome (GBS), the most common cause of acute, flaccid neuromuscular paralysis, is autoimmune-mediated destruction of nerves in the peripheral nervous system, with a global incidence rate of about 1-2 per 100,000 person-years and a mortality rate of 3-10% despite receiving medical care [1]. GBS typically presents progressive weakness and sensory impairments that start in the distal extremities and progress proximally toward the arms and cranial muscles [1]. Commonly recognized manifestations include hyporeflexia, ascending symmetrical muscle weakness of paresthesia, facial palsy, ophthalmoplegia, and ataxia [1]. GBS is diagnosed clinically, which can be supported with additional tests such as cerebrospinal fluid (CSF) analysis and electrophysiological studies [1]. Early detection of GBS is critical, given it can rapidly progress into respiratory failure, requiring mechanical ventilation [1]. Additionally, GBS can affect the autonomic nervous system, potentially leading to cardiac arrhythmias and unstable blood pressure, both of which are significant contributors to GBS-related mortality [1].

GBS usually occurs after an infectious process, often a prior gastrointestinal or respiratory infection, in which the immune response generates antibodies that cross-react with gangliosides, such as GM1 and GQ1b, at nerve membranes, resulting in functional nerve damage [2]. The most common pathogen triggering molecular mimicry that leads to GBS is *Campylobacter jejuni*, a cause of acute gastroenteritis [3]. Other pathogens implicated in the triggering of GBS are influenza, cytomegalovirus (CMV), Epstein-Barr virus (EBV), human immunodeficiency virus (HIV), chikungunya, Zika virus, and *Mycoplasma pneumoniae* [4]. There has been an equally important potential link between GBS and vaccinations, including influenza, MMR (measles, mumps, and rubella), hepatitis B, and diphtheria [5]. Additionally, there has been emerging evidence suggesting an association between both viral vector-based and mRNA-based SARS-CoV-2 (COVID-19) vaccines and GBS, although this link remains controversial [5].

In May 2023, the United States Food and Drug Administration (FDA) approved two novel vaccines for adults ages 60 years and above to prevent respiratory syncytial virus (RSV)-associated lower respiratory disease: RSVPreF3 (Arexy, GSK) and RSVpreF (Abrysvo, Pfizer) [6]. RSVPreF3 (Arexy, GSK) is an ASO1E-adjuvanted recombinant stabilized perfusion F protein vaccine, while RSVpreF (Abrysvo, Pfizer) is a recombinant stabilized preF vaccine [6]. Clinical trials for both vaccines reported an occurrence of inflammatory neurological events in three adults within 42 days post-vaccination among their respective cohorts: 17,922 adults for GSK and 20,224 adults for Pfizer [6]. Notably, each vaccine trial included a single case of GBS [6]. Although it is a rare finding, we present a case of a patient who developed GBS two weeks after receiving his RSV vaccination with coadministration of the influenza vaccine, highlighting the need to further investigate its relationship with neurological pathophysiology.

## Case presentation

A 60-year-old Caucasian male with a medical history of peripheral artery disease, hyperlipidemia, hypertension, and trigeminal neuralgia presented to the emergency department (ED) with a chief complaint of progressive tingling that developed into numbness. Two weeks prior to the presentation, he experienced new-onset tingling in both of his legs accompanied by nausea, vomiting, constipation, metallic taste, body aches, and lower back pain. At that time, he was evaluated in the ED, where he had a normal neurological exam and an unremarkable computed tomography (CT) scan of his chest, abdomen, and pelvis. He was diagnosed with exacerbation of sciatica and discharged. Five days later, the patient's tingling in the lower extremities subsequently transformed into numbness, causing stumbling and difficulty walking. The symptoms then ascended to his hands and mouth, prompting his second ED visit and subsequent admission.

Approximately three weeks before these symptoms appeared, the patient received Pfizer’s COVID-19 vaccine booster, followed by his first-ever administration of both the Flucelvax® Quadrivalent influenza vaccine and Pfizer’s newly approved RSV vaccine five days later. He reported no adverse reactions from previous COVID-19 vaccinations. His diet had recently been limited to rice cakes due to ongoing constipation and indigestion. He experienced one episode of non-bloody diarrhea after taking bisacodyl for constipation and noted a weak urinary stream without saddle anesthesia for the past three days. He did not report consumption of poultry or meat for several weeks, no home canning activities and his wife exhibited no similar symptoms despite consuming the same foods as the patient.

In the ED, the patient presented with an elevated blood pressure of 148/93 mmH; vital signs were otherwise within normal limits. The electrocardiogram demonstrated normal sinus rhythm. The neurological assessment showed a normal cranial nerve exam, absent deep tendon reflexes in the bilateral Achilles, patellar, bicep, and triceps tendons, with decreased sensation across all extremities. The motor exam showed bilateral lower extremities with 0/5 strength while bilateral upper extremities were 1/5 strength. Physical examination showed mild tenderness in the lower abdominal quadrant upon deep palpation, otherwise unremarkable. Laboratory tests showed abnormalities of a white blood cell (WBC) count of 11x103/uL, sodium of 135 mEq/L, blood urea nitrogen of 21 mg/L, creatinine of 1.06 mg/dL, and magnesium of 2.6 mg/dL (Tables 1-3). Cerebrospinal fluid (CSF) examination revealed clear fluid and albuminocytologic dissociation, with an elevated protein of 84 mg/dL and normal leukocyte count of WBC 2 cells/uL (Table 4). Viral panel tests for Influenza A and B, RSV, and COVID-19 were negative. A CT scan of the brain without contrast showed no abnormalities.

**Table 1:** Complete blood count laboratory results WBC: White blood cells, RBC: Red blood cells, MCV: Mean corpuscular volume, MCH: Mean corpuscular hemoglobin, MCHC: Mean corpuscular hemoglobin concentration, RDW: Red cell distribution width

Labs	Result	Normal Reference Range
WBC	11.0 10^3^ / uL	4.3-10.6 10^3^ / uL
RBC	5.52 10^6^ /uL	4.30-5.80 10^6^ /uL
Hemoglobin	16.6 g/dL	13.3-17.0 g/dL
Hematocrit	47.0%	40.3-50.3%
MCV	85.1 fL	81.4-99.0 fL
MCH	30.1 pg	26.8-32.9 pg
MCHC	35.3 g/dL	31.4-34.9 g/dL
RDW	11.9%	11.6-14.9%
Platelets	192 10^3^ / uL	132-337 10^3^ / uL
Neutrophils Absolute	8.2 10^3 ^/ uL	1.5-7.5 10^3 ^/ uL
Lymphocytes Absolute	1.7 10^3^ /uL	0.7-3.6 10^3^ /uL
Monocytes Absolute	0.9 10^3^ /uL	0.2-1.1 10^3^ /uL
Eosinophils Absolute	0.08 10^3^ /uL	0.00-0.50 10^3^ /uL
Basophils Absolute	0.1 10^3^ /uL	0.0-0.1 10^3^ /uL
Neutrophils Relative	74.7%	41.7-78.9%
Lymphocytes Relative	15.1%	10.8-45.9%
Monocytes Relative	8.6%	3.7%-15.0%
Eosinophils Relative	0.7%	0.0-5.7%
Basophils Relative	0.5%	0.0-1.5%
IG Relative	0.4%	0.0-3.0%
Abs Neutrophil Count	8, 217 Cells/mm^3^	1,500-8,000 Cells/mm^3^

**Table 2:** Basic metabolic panel laboratory results eGFR: Estimated glomerular filtration rate

Labs	Result	Normal Reference Range
Sodium	135 mEq/L	136-144 mEq/L
Potassium	4.1 mEq/L	3.5-5.1 mEq/L
Chloride	98 mEq/L	101-111 mEq/L
CO2	26 mEq/L	22-32 mEq/L
Blood urea nitrogen	21 mg/dL	8-20 mg/dL
Glucose	100 mg/dL	70-99 mg/dL
Calcium	8.8 mg/dL	8.5-10.1 mg/dL
Creatinine	1.06 mg/dL	0.55-1.02 mg/dL
Anion gap	11 mEq/L	8-17 mEq/L
eGFR Male	>60.0 mL/min/1.73 m^2^	>= 60.0 mL/min/1.73 m^2^

**Table 3:** Additional laboratory results

Labs	Result	Normal Reference Range
Magnesium	2.6 mg/dL	1.8-2.5 mg/dL
Total creatine kinase	101 U/L	49-397 U/L
Myelin basic protein	<2.0 mcg/L	≤4.0 mcg/L

**Table 4:** Cerebrospinal fluid (CSF) laboratory results

Labs	Result	Normal Reference Range
Glucose	61 mg/dL	40-70 mg/dL
Protein	84.0 mg/dL	15.0-45.0 mg/dL
Color	Colorless	Colorless
Red blood cells	2 cells/uL	0-5 cells/uL
White blood cells	2 cells/uL	0-5 cells/uL
Appearance	Clear	Clear
Culture	Culture: no anaerobes isolated in 5 days No Organisms seen	Culture: no organisms

The patient’s clinical presentation and CSF findings with elevated protein levels were consistent with a diagnosis of GBS. The patient was started on immunoglobulin (IVIg) therapy for a total of five days. Respiratory vitals were monitored closely, and fortunately, his negative inspiratory force (NIF) remained within normal limits for the duration of his hospitalization, ranging from -60 to -85 cm H2O and never dropping below -30 cm H2O, so intubation and ventilatory support were not required during his stay. The patient was discharged upon completion of IVIg therapy with slight improvements in his symptoms and was instructed to follow up with outpatient neurology in two weeks. During this subsequent visit, he reported a continued lack of sensation in his hands and feet, but notably, his patellar reflex had returned for the first time since the onset of GBS. As a result, he was scheduled for an additional round of IVIg therapy at 400 mg/kg for five doses and further evaluations with electromyography and magnetic resonance imaging (MRI) as an outpatient.

## Discussion

The link between GBS and influenza vaccination was first noted in 1976 during the United States’ pandemic swine flu vaccination program, where an eightfold increase in GBS cases was observed after vaccinating about 40 million people [7]. However, subsequent studies indicated the risk of GBS with later seasonal influenza vaccines was minimal [7]. The highest risk period is within the first two to three weeks after vaccination, with an estimated incidence of one to two cases per one million vaccinations [7]. While this establishes the overall safety of vaccination, it is crucial to investigate its administration in conjugation with other vaccines that may pose a risk of GBS, such as the novel RSV vaccine. In an analysis of Pfizer’s RSV vaccine trials involving 20,255 participants, one case of GBS with symptoms developed fourteen days after vaccination was reported [6]. Similarly, within GSK’s RSV vaccine clinical trials encompassing 17,922 participants, there was one case of GBS with symptoms manifesting nine days post-vaccination [6].

As in our patient’s case, many individuals receive the RSV and influenza vaccines at the same time, given the Centers for Disease Control and Prevention (CDC) considers co-administration to be safe [8]. However, there is limited data on this combination due to the relatively recent introduction of RSV vaccines. The existing evidence regarding whether there may be an increased reactogenicity with coadministration of RSV and influenza vaccines is mixed [9]. This highlights the importance of exploring the potential summative effects of these vaccines on developing clinically significant outcomes, such as GBS, which will help guide the safe practices for the coadministration of RSV vaccines. Currently, the CDC states that there is no minimum waiting period between the influenza, RSV, and COVID-19 vaccines [8]. However, clinical pharmaceutical experts have recommended spacing out the RSV vaccine from the influenza and COVID-19 vaccines by 1-2 weeks to minimize side effects [10]. Implementing a reasonable waiting period between the RSV and influenza vaccines and conducting research to assess if this approach reduces side effects may serve as a precautionary measure to reduce the risk of GBS.

Early recognition of GBS symptoms is imperative given that they can present subtly and affect both the peripheral and autonomic nervous systems. Our patient initially presented to the ED with a metallic taste, back pain, lower extremity tingling, and constipation-which were collectively subtle manifestations of GBS. Roughly, 0.6-2% of GBS cases report taste impairment; however, this number is likely underestimated [11]. This was evidenced by a study using electrogustometry to conduct taste threshold tests, which revealed taste impairment in 60% of GBS patients, but only 20% of these patients reported experiencing dysgeusia [11]. This may be due to the chorda tympani branch of the facial nerve being primarily composed of small myelinated fibers, while GBS more likely targets large myelinated fibers [11]. Dysgeusia may be neglected clinically, and more standardized tests should be conducted to quantify this symptom so providers are more aware of its association with GBS [12]. Moreover, our patient’s lower back pain and lower extremity tingling were attributed to sciatica. It is important to note that 54-89% of patients experience pain, including paresthesia and back pain, which presents before the development of muscle weakness in about one-third of GBS cases [3]. Additionally, the patient’s constipation may have been a manifestation of autonomic nervous system dysfunction from GBS, which was treated with enema and diet modification. Unfortunately, the patient returned one week later with worsening neurological symptoms and is still experiencing residual deficits. This possible initial oversight in diagnosis could account for the delayed initiation of IVIg treatment. Studies have indicated that failure to consider GBS on the initial assessment was associated with higher risks of residual weakness and intubation, and neurologists recommend prompt treatment even in mild cases to avoid deterioration [13]. In a study involving 32 out of 41 GBS patients who experienced a delay in the initiation of treatment, the predominant factor was the consideration of alternative etiologies for presenting symptoms [13]. Notably in this study, 11 patients were thought to have orthopedic issues, and six were considered to have nutritional deficiencies, as seen in this patient’s case as well [13]. Although back pain and constipation can be common symptoms in the ED with a variety of possible and often more common etiologies, such as chronic back pain managed with opioids, GBS should be considered as a differential diagnosis in the appropriate clinical scenarios when suspicion is high, such as in cases with unexplained neurological symptoms after recent vaccine administrations within a short timeframe. This case demonstrates the challenge in diagnosing GBS given its variable presentation. Early and accurate identification of GBS is vital for timely intervention as it can significantly affect patient outcomes.

## Conclusions

In this report, we present a case of a 60-year-old man who developed GBS two weeks after simultaneously receiving Pfizer's new RSV vaccine and the influenza vaccine for the first time. GBS significantly impacts a patient's ability to perform tasks in their everyday life, as it did for our patient by affecting his ability to ambulate. In light of this, it is imperative to examine the association with and effects of vaccines and vaccine co-administration with respect to the pathogenesis of GBS. Although the CDC considers the coadministration of these vaccinations to be safe, more studies are needed to further evaluate the incidence of GBS following the coadministration of these vaccinations.
